# High-Translucency Zirconia Following Chemical Vapor Deposition with SiH_4_: Evidence of Surface Modifications and Improved Bonding

**DOI:** 10.3290/j.jad.b3801051

**Published:** 2023-01-12

**Authors:** Jaiane Bandoli Monteiro, Pedro Henrique Condé Oliveira Prado, Gabriela Ribeiro Zucco, Tiago Moreira Bastos Campos, João Paulo Barros Machado, Vladimir Jesus Trava-Airoldi, Renata Marques de Melo

**Affiliations:** a PhD Student, Postgraduate Program in Restorative Dentistry (Prosthodontics Unit), São Paulo State University (UNESP), Science and Technology Institute, São José dos Campos, São Paulo, Brazil. Contributed to the study design, performed experiments, statistical analysis, wrote the manuscript.; b Master’s Student, Postgraduate Program in Restorative Dentistry (Prosthodontics Unit), São Paulo State University (UNESP), Science and Technology Institute, São José dos Campos, São Paulo, Brazil. Performed experiments, wrote the manuscript; c Master’s Student, Postgraduate Program in Restorative Dentistry (Prosthodontics Unit), São Paulo State University (UNESP), Science and Technology Institute, São José dos Campos, São Paulo, Brazil. Performed experiments.; d Researcher, Physics Department, Aeronautics Technological Institute (ITA), São José dos Campos, São Paulo, Brazil. study idea, study design, performed experiments, data analysis.; e Researcher, National Institute for Space Research, Associated Laboratory of Sensors and Materials, São José dos Campos, São Paulo, Brazil. study design, data analysis.; f Researcher, National Institute for Space Research, Associated Laboratory of Sensors and Materials, São José dos Campos, São Paulo, Brazil. Study design, proofread the manuscript.; g Professor, Department of Dental Materials and Prosthodontics, São Paulo State University (UNESP), Institute of Science and Technology, São José dos Campos, São Paulo, Brazil. study idea, study design, proofread the manuscript.

**Keywords:** dental ceramics, zirconia, silicon, aging, bond strength.

## Abstract

**Purpose:**

To evaluate the effect of plasma-enhanced chemical vapor deposition (PECVD) with silicon hydride (SiH_4_) at different times on HT-zirconia surface characteristics and bonding of composite cement before and after thermocycling.

**Materials and Methods:**

Blocks of HT zirconia were obtained, polished, sintered and divided into five groups, according to PECVD time (n = 31): Zr-30 (30 s), Zr-60 (60 s), Zr-120 (120 s) and Zr-300 (300 s). The control group (Zr-0) did not receive PECVD. X-ray diffraction (XRD), Fourier-transform infrared spectroscopy (FTIR), energy dispersive spectroscopy (EDS) in conjunction with field-emission scanning electron microscopy (FE-SEM), x-ray photoelectron spectroscopy (XPS), goniometry, and profilometry tests were used for chemical and topographic characterization. Monobond N silane (Ivoclar Vivadent) was applied to the surface, and a cylinder of composite cement (Variolink N) was made (3 x 3 mm). Half of the specimens of each group were stored for 24 h or subjected to thermocycling (6 x 10^3^ cycles). A shear bond strength (SBS) test was performed. Results were subjected to one-way ANOVA and Tukey’s tests (α = 0.05).

**Results:**

For experimental groups, XPS showed that formation of Si-O bonds contributed to increased surface free energy (SFE). FE-SEM and EDS showed that the longer the deposition time, the greater the amount of silicon on the surface. Zr-60 and Zr-300 presented higher and lower surface roughnesses, respectively. The silicon penetrated the microstructure, causing higher stress concentrations. The bond strength to composite cement was improved after all PECVD deposition times.

**Conclusion:**

The PECVD technique with SiH_4_, associated with chemical treatment with primer based on silane methacrylate, is a solely chemical surface treatment capable of maintaining bonding between composite cement and HT zirconia.

The constant search for fully ceramic restorative materials that combine esthetics and high strength, along with the evolution of CAD/CAM (computer-aided design/computer-aided manufacturing) systems, has contributed to the development of Y-TZP zirconia (yttria tetragonal zirconia polycrystal).^11,29^ Zirconia is a polymorphic material, existing in three distinct crystallographic forms: monoclinic, tetragonal, and cubic.^15^ For the stabilization of zirconia in its tetragonal phase, 3% mol yttrium oxide was included in its composition,^34^ guaranteeing high flexural strength, high fracture toughness, and better chemical stability.^2,34^

The clinical indication of ceramic systems is based on the mechanical and optical properties of the materials. Therefore, Y-TZP zirconia has become applicable in indirect ceramic restorations, such as full crowns and fixed dental prostheses,^11,24^ where veneering ceramics must also be applied for suitable esthetics.^47^ Fractures and chipping are the most common problems of the veneered zirconia cores,^30,37^ mostly due to interfacial stress-generating thermal coefficient mismatches and zirconia’s low thermal conductivity.^14,47^

High-translucency (HT) zirconia is a second-generation 3-YTZP with little gain in translucency. It is therefore indicated for monolithic posterior restorations,^30,49,55^ mainly in situations where the interocclusal space is limited, since the occlusal thickness can be reduced to 0.5 mm while maintaining enough resistance to withstand occlusal loads.^31,32,39,41^ However, HT zirconia was also explored for full-contour anterior restorations with extrinsic characterization.^43^ In general, the mechanical properties of first and second generation zirconias are alike, but the latter presents fewer porosities and fewer alumina additives.

The clinical issue arising from the use of monolithic zirconia restorations is the difficulty in achieving strong adhesion with dental cements and loss of retention at the restoration interface.^17,39,47^ Several reasons may explain this type of failure, such as its microstructure (a polycrystalline material without a glassy phase, which makes it acid resistant and thus non-etchable) or its hydrophobic nature, which causes low wettability of the zirconia surface by the adhesive cements.^29^

Currently, research is being done on several in-vitro and clinical techniques to solve the problem of bonding composite cements to ceramics. Most of them are focused on micromechanical and/or chemical modifications of the surfaces of those ceramics, opening a variety of treatment options^23^ in search of durable bonding of the ceramic with composite cements and dental tissue.^20,50^ However, even with the combination of techniques, it has not been possible to obtain a long-term bond of composite cement to zirconia.^9,23^ There are reports in the literature showing that the association between mechanical treatment (tribochemical silica sandblasting) and chemical conditioning (with primer) provided the highest zirconia bonding efficacy, even with hydrothermal aging.^19^ Favorable chemical bonding to silica-coated zirconia can be achieved using the phosphate-based functional monomer 10-MDP,^8^ and/or silane methacrylate.^3^

To date, there has been no agreement about a non-destructive, effective surface treatment to obtain optimal bond strength of composite cement to HT zirconia. Therefore, it is necessary to seek treatments with the ability to chemically functionalize the zirconia surface. Ideally, this would enable an adhesive bond without causing structural and mechanical damage, resulting in strong chemical adhesion and increased bond strength of the composite cement to the ceramic, facilitating the long-term clinical success of such zirconia restorations.

Studies have shown that plasma-enhanced chemical vapor deposition (PECVD), consisiting of a deposition of silicon particles from the gaseous state to a solid state on the material surface, results in chemical adhesion and increases bond strength between substrates.^6,13,22,33^ Putting it another way, a silica-like surface layer rich in binding sites for silanes can produce equal or higher bonding efficacy than silicatization of zirconia surfaces. When associated with primer application based on MDP or silane methacrylate, it promotes long-term adhesion of the cementing agent,^3,4,9^ which is desirable in many clinical situations, such as in teeth with minimal preparations. When bond strengths were not improved after PECVD, the main issue was defining the best parameters for the deposition (eg, time and layer thickness).^42^ Thicker seed layers tend to be chemically bound to the zirconia only near its surface, leading to a reduction in bond strength. Furthermore, PECVD studies have failed to include aging of the specimens, overlooking the fact that a strong chemical bond should also be durable and survive long-term fatigue.^13,47^

Therefore, the aim of this study was to characterize the surface topography of zirconia after the use of PECVD with SiH_4_ gas for different durations, as well as to evaluate the chemical influence of PECVD on the zirconia surface, and examine the chemical bonding receptiveness of composite cement in terms of shear bond strength, before and after aging. The null hypotheses tested were that: (1) the proposed treatment would not chemically modify the zirconia, and (2) the longest application times of SiH_4_ via PECVD would not increase shear bond strength of composite cement before and after aging.

## Materials and Methods

### Specimen Preparation

The largest (39 x 19 x 15.5 mm) non-sintered zirconia-based ceramic blocks (VITA YZ HT zirconia, Vita Zahnfabrik; Bad Säckingen, Germany) available for milling were prepared by means of a diamond disk at 100 rpm in a cutting machine (IsoMet 1000, Buehler; Lake Bluff, IL, USA) under water cooling to obtain smaller specimens (N = 155) (4.6 x 3.7 x 3 mm). The specimens were standardized using silicon-carbide papers (Norton Saint Gobain; São Paulo, Brazil) with decreasing granulation of #400, #800, and #1200 in a polishing machine (EcoMet 250 Grinder Polisher, Buehler) under water cooling.

After being cut and polished, all blocks were cleaned with isopropyl alcohol for 5 min in an ultrasonic bath (Cristófoli Ultrasonic Washer; Campo Mourão, Brazil). All specimens were sintered according to the manufacturer’s instructions (VITA Zyrcomat, Vita Zahnfabrik; final sintering temperature, 1450°C, for approximately 4 h and 40 min). After being sintered, all blocks were again cleaned with isopropyl alcohol in an ultrasonic bath for another 5 min.

### Plasma-enhanced Chemical Vapor Deposition (PECVD)

**Fig 1:**
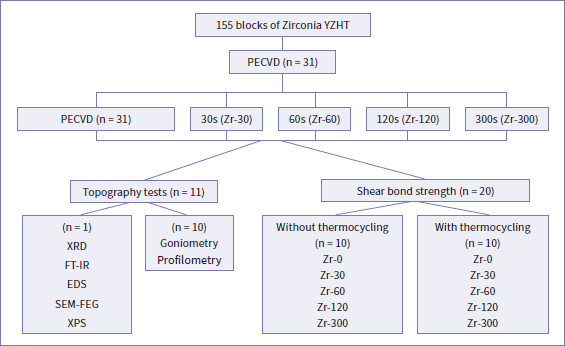
Flowchart of experimental procedures.

Before PECVD was performed, the samples were stored in an oven (Olidef; Ribeirão Preto, São Paulo, Brazil) at 37ºC for 24 h. For the chemical deposition of the SiH_4_ gas on the surfaces of the samples, an in-house vacuum reactor was used. After several pilot tests, the deposition duration seemed the most critical parameter for good adhesion. Thus, the specimens were randomly assigned to groups depending on the duration of PECVD application: Zr-30 (30 s), Zr-60 (60 s), Zr-120 (120 s), and Zr-300 (300 s). The control group (Zr-0) did not receive deposition (0 s) (Fig 1).

The vacuum reactor chamber is equipped with an inlet tube for gases and an outlet tube with a vacuum pump, which eliminates remaining unreacted gases from the chamber’s interior. Prior to the experiment, the chamber was bombarded with argon to clean both the chamber and zirconia samples under the following conditions: pressure of approximately 2.4 x 10^-3^ Torr and low voltage source (2 kV) for 5 min. The conditions for depositing SiH_4_ gas were a high voltage source (10 kV), pressure of 2.4 x 10^-3^ Torr, and a pulse of 5 μs. A potential difference was generated, and a plasma cloud arose to increase the gas reactivity. The pressure established inside the chamber, the applied voltage, the reactor configuration, and the thermodynamic equilibrium conditions led to greater effectiveness and homogeneity of the silicon and hydrogen ions to be deposited on the surfaces of the zirconia samples. The thermodynamic equilibrium conditions contributed to the breakdown of SiH_4_ bonds into silicon and hydrogen ions and the bombardment of the zirconia surface. The deposition duration was controlled with a digital timer (Unilab; São Paulo, Brazil).

### Zirconia Surface Analyses

One specimen each per group was evaluated by x-ray diffraction (XRD), Fourier-transform infrared (FTIR) spectroscopy, scanning electron microscopy with a field emission gun (FE-SEM), SEM with energy-dispersive x-ray spectroscopy (EDS), and x-ray photoelectron spectroscopy (XPS) (Fig 1). X-ray diffraction (XRD) (X’pert Powder model, PANalytical; Westborough, MA, USA) was performed at 10°-90º, with a scan step of 10.16 s, at a 0.017º step size, with CuKα radiation. The infrared spectra were acquired by FTIR spectroscopy with a universal attenuated total reflectance sensor (FTIR-UATR) (PerkinElmer Spectrum, Frontier model; Waltham, MA, USA). The FTIR spectrum was an average of 16 scans at a speed of 2 s per scan at a range of 500-4000 cm^-1^. The resolution of the spectrometer was set at 4 cm^-1^ (Spectrum Search Plus Program, PerkinElmer). EDS was performed by spectrometry with an energy-dispersive x-ray device (Bruker Nano 410; Berlin, Germany) coupled to an SEM (Inspect S50, FEI; Brno, Czech Republic; Esprit 1.9 software, Bruker). FE-SEM (Tescan, Mira 3; Brno, Czech Republic), secondary electron (SE) and back-scattered scanning electron (BSE) detectors were used. The samples were placed on a circular stub to obtain images at magnifications of 3000X, 5000X, 10,000X, and 15,000X. XPS analyses were performed in a spectrometer (Kratos Axis Ultra DLD; Nanuet, NY, USA) with an Al Mono monochromatic source (1486.6 eV) and 120 W power. The survey XPS spectra were recorded with a pass energy (PE) of 160 eV and spectra in high resolution with a PE of 40 eV. The spectra were calibrated relative to the O-1s peak at 529.2 eV, with a charge neutralizer at the ON position. All the measurements were performed in ultra-high vacuum at < 10^-7^ Pa pressure, 15 kV acceleration voltage, and 10 mA power emission.

In total, 10 specimens per group were used for optical profilometry analysis and contact angle measurements. Surface roughness was analyzed by means of an optical profilometer (Wyko NT 1100, Veeco; Plainview, NY, USA; Wyko Vision 32 software, VSI mode, Veeco). Measurements of the three-dimensional parameters were performed at magnifications of 20.5X, in a 300 x 230-μm area. Data were plotted and analyzed using one-way ANOVA (α = 0.05). The total surface free energy (SFE) and SFE of the polar and dispersive solids (in mN/n) were calculated from the mean contact angle (n = 10) by means of a goniometer and DROPimage Advanced software (Ramé Hart; Mountain Lakes, NJ, USA) using the sessile drop technique with distilled water and diiodomethane at room temperature.

### Shear Bond Strength (SBS) Test

The composition, manufacturer, batch, and expiration date of the coupling agents/luting composites are listed in Table 1.

**Table 1:** Materials used in the study

Material	Manufacturer	Composition	Batch No.	Validity
Zirconia YZ HT	Vita Zahnfabrik; Bad Säckingen, Germany	ZrO_2_, Y2O_3_, Al_2_O_3_, SiO_2_, Fe_2_O_3_, Na_2_O	62700	Indeterminate
Monobond N	Ivoclar Vivadent; Schaan, Liechtenstein	Alcohol solution of silane methacrylate, phosphoric acid methacrylate, sulphide methacrylate	U29879	07/2017
Variolink N Base	Ivoclar Vivadent	Bis-GMA, urethane dimethacrylate, triethylene glycol dimethacrylate, barium glass, ytterbium trifluoride, Ba-Al-fluorosilicate glass, spheroid mixed oxide, initiators, stabilizers, pigments	V00666	04/2018
Variolink N Catalyst			U48611	04/2018

A sample size calculation for shear bond strength data was made for a test power of 80%. 100 samples (n = 10) were used for SBS testing: 50 for testing at 24 h and 50 for testing after thermocycling were embedded in autopolymerizable acrylic resin (Jet, Clássico Dental Articles; São Paulo, Brazil) in a PVC cylinder (Tigre; São Paulo, Brazil). A thin layer of silane (Monobond N, Ivoclar Vivadent; Schaan, Liechtenstein) was applied with a microbrush onto the zirconia surface for 10 s, allowing the material to react for 60 s. A dual-curing composite cement cylinder (internal diameter, 3 mm; height, 3 mm) (Variolink N Base and Catalyst, Ivoclar Vivadent) was built up on the surface of each sample with the aid of Tygon tubing. The cement was injected via a syringe and photopolymerization was carried out with an LED curing light (Valo, Ultradent; South Jordan, Utah, USA) at an intensity of 1000 mW/cm^2^ and a wavelength of 395 to 480 nm for 20 s per interface, simulating the occlusal, buccal, and lingual aspects, totaling 60 s. The specimens were stored in distilled water in an incubator (Olidef) at 37ºC for 24 h.

SBS testing was performed in a universal testing machine (EMIC DL 1000, EMIC; São José dos Pinhais, Paraná, Brazil; 0.45 mm diameter wire, 50 kgf load cell, speed 1 mm/min). Half of the samples from each group were tested 24 h after cementation, while the other half were subjected to 6000 thermal cycles (5ºC to 55ºC) in a thermocycler (Termocycle, Biopdi, São Carlos, SP, Brazil) before testing. The thermocycled groups were named according to the deposition duration: Zr-0 t (control), Zr-30 t (30 s), Zr-60 t (60 s), Zr-120 t (120 s), and Zr-300 t (300 s) (Fig 1).

After the SBS test, the zirconia surface was evaluated under a binocular stereomicroscope (Discovery V20, Carl Zeiss; Göttingen, Germany) to determine the interfacial mode of failure (adhesive or predominantly adhesive at the interface between cement and zirconia; cohesive in cement or zirconia; and mixed: adhesive failure plus cohesive failure in cement). The SBS data were obtained in MPa according to the formula:

SBS = force/bonding area

in which the force is in N and the bonding area is given in mm^2^, mathematically expressed by π (3.14) multiplied by r^2^ (radius of the circumference = 1.5^2^ = 2.25). The bond strengths were analyzed with the Shapiro-Wilk test to verify the assumption of normality of the data (p>0.1). One-way ANOVA followed by Tukey’s post-hoc multiple-comparison test was used to compare SBS results. Significance was set at 0.05.

## Results

### Surface Analyses

**Fig 2:**
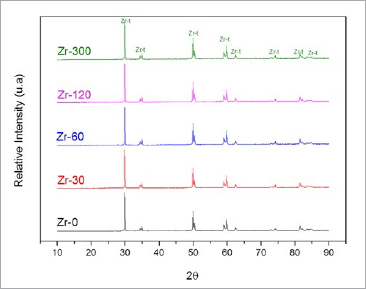
X-ray diffractograms of Zr-0, Zr-30, Zr-60, Zr-120, and Zr-300. The peaks signaled by Zr-t correspond to tetragonal phases.

**Fig 3:**
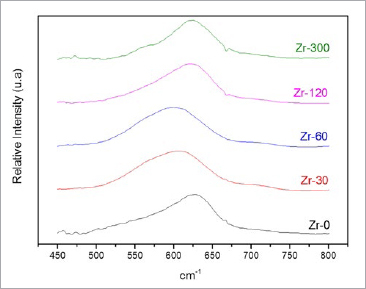
Representative FTIR spectra of control (Zr-0) and experimental groups (Zr-30, Zr-60, Zr-120, and Zr-300).

**Fig 4:**
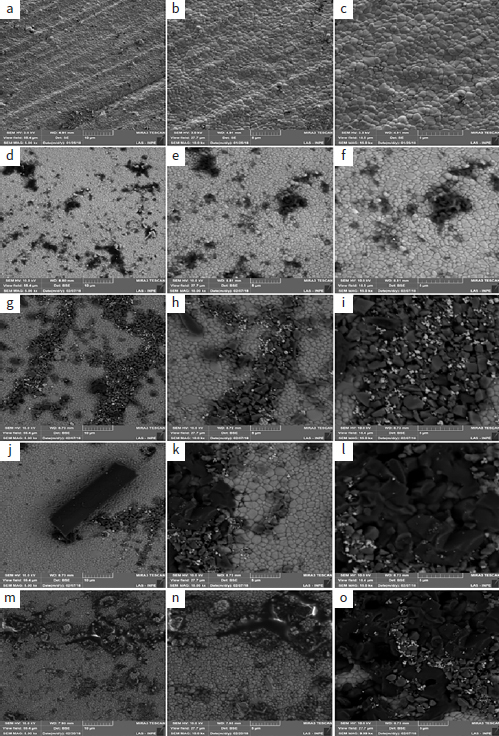
Representative micrographs of the zirconia surface (original magnifications of 5000X, 10,000X, and 15,000X). (a-c) Zr-0 (after the polishing and without PEVCD) and after PECVD (d-f) with 30 s (Zr-30), (g-i) 60 s (Zr-60), (j-l) 120 s (Zr-120), and (m-o) 300 s (Zr-300).

**Fig 5:**
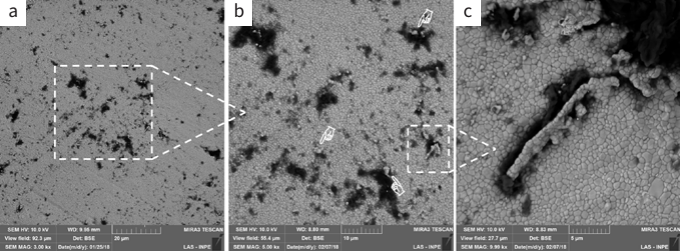
Micrographs of sample surfaces (a: 3000X, b: 5000X, and c: 10,000X) treated with Zr-120 PECVD showing silicon penetration and the beginning of zirconia grain detachment (pointers).

**Fig 6:**
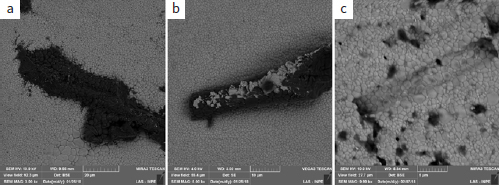
Micrographs of sample surfaces at different stages of silicon cylinder detachment: a: beginning, Zr-60 (60 s – 3000X); b: mid-way, Zr-120 (120 s – 5000X); end, c: Zr-300 (300 s – 10,000X) .

**Fig 7:**
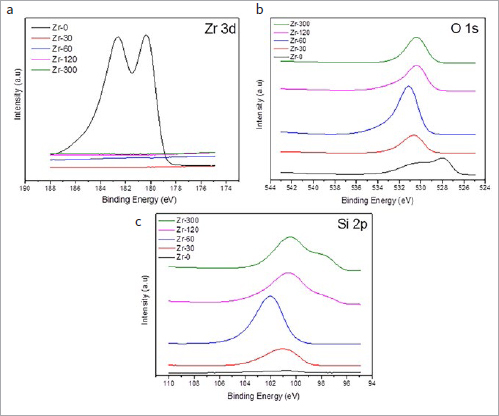
XPS spectra of (a) Zr-3d, (b) O-1s, and (c) Si-2p spectra for the control and experimental groups.

XRD, FTIR spectroscopy, EDS, FE-SEM, and XPS results are presented in Figs 2–7.

Figure 2 shows the x-ray diffractograms of the control and experimental samples. The XRD spectra were identical for the different PECVD conditions and control group, showing that all diffraction peaks presented could be attributed to the plane of the tetragonal ZrO_2_ phase.^26,40,46^ These results showed that the interaction between silicon and zirconia did not form a crystalline phase.

In the FTIR spectrogram (Fig 3), all samples had a broad band between 500 cm^-1^ and 700 cm^-1^. The strong absorption peaks on these spectra correspond to the Zr-0 vibrational modes. The shape and position of the center of the band shifts depend on the silicon deposition durations. For Zr-30 and Zr-60, there was a shift toward lower frequencies, which means that the longer the exposure time, the higher the compressive stress on the zirconia, and the more the band tends to move to the left. This compressive stress can be caused by the penetration of silicon between the zirconia grains (Fig 5).

For Zr-120 and Zr-300, these bands returned to frequencies similar to Zr-0. This displacement can be associated with the stress that occurred during the PECVD process. With the detachment of the zirconia grains (Fig 6), compression stress relief leads to the change of position of the band, returning to its original position. In other words, we affirm that the bands for Zr-120 and Zr-300 returned to similar frequencies of Zr-0 because, after applying PECVD for 120 and 300 s, silicon accumulated into the defects of zirconia, filling the spaces between the grains and causing the detachment. This detachment, in turn, caused a decrease of compressive stress on the surface.

Chemical analysis by EDS (Table 2) showed that with increasing deposition durations, the amount of silicon present on the zirconia surface also increased. There was also an increase in oxygen. Figure 4 shows the micrographs of representative samples of all groups (control and experimental). In the surface micrographs of the Zr-0 sample, the zirconia grains are well-delimited, rounded, and homogeneous (Figs 4a to 4c). In the experimental groups, dark spots were seen under the BSE detector. EDS showed the presence of silicon, which has an atomic weight (28.0855 u) lower than that of zirconia (91.224 u).

**Table 2:** Composition in weight percent (%) of control and experimental groups by EDS analysis

Groups (weight %)					
Chemical element	Zr-0	Zr-30	Zr-60	Zr-120	Zr-300
Zirconia	73.1	72.7	70.5	68.8	67.4
Oxygen	20.3	20.9	22.7	23.8	25.5
Yttrium	6.6	6.1	6.0	6.1	5.8
Silicon	–	0.3	0.8	1.4	1.4

Longer deposition durations are responsible for the greater number of silicon clusters present on the surface of the zirconia. The penetration of silicon covered the defects from processing, causing conformational change of zirconia (resembling irregular polyhedral forms) (Figs 4g-4l) and compression stress on the surface; therefore, the infrared band was displaced (Zr-30 and Zr-60) (Fig 3).

For Zr-120 and Zr-300, there was a critical volumetric increase of zirconia grains until detachment (Fig 5) and detachment of a stick-like silicon structure (Figs 6b and 6c). The detachment can be better visualized in Fig 6 and always occurred near processing defects. The 60-s deposition (Zr-60) caused silicon penetration and cylinder formation (Fig 6a), which became clear and apparently detached from the silicon grains with 120-s deposition (Zr-120) (Fig 6b), becoming fully detached from the surface of the zirconia with 300-s deposition (Zr-300) (Fig 6c).

Figure 7 shows XPS spectra from control and experimental samples. Only the Zr-0 group (without PECVD) exhibited the Zr-3d spectrum, where peaks were centered at approximately 180 eV, indicating peaks related to Zr-O-Zr bonds (ZrO_2_). It was not possible to identify Zr-O-Zr bonds on the surface of any sample of the experimental groups (Fig 7a). For the O-1s spectra, the peaks were centered at approximately 526-529 eV for Zr-0 and 529-533 eV for all experimental groups (Fig 7b). Finally, for the Si-2p spectra, a centered peak was attributed to Si substrate at around 99 eV^18^ for both Zr-120 and Zr-300 (Fig 7C). The Si-2p peak between 102 and 103 eV for all experimental groups indicates Si-O bonds (SiO and SiO_2_) (Fig 7c).^20^

The surface roughness data are described in Table 3. One-way ANOVA showed statistically significant differences between all groups (p < 0.05). In Tukey’s test, Zr-0 (with a grooved surface) and Zr-120 were not statistically significantly different. The samples of zirconia with a deposition time of 60 s (Zr-60) showed greater superficial roughness (376.1 ± 16.8 nm) in comparison with the other groups. The group that received the longest deposition time (Zr-300) presented the smoothest and most homogeneous surface, with the lowest values of surface roughness (241.0 ± 12.9 nm).

**Table 3:** Means and standard deviations (SD) in nm, confidence intervals (CI) of surface roughness values (Ra) of control and experimental groups

Groups	Mean and SD (nm)	95% CI
Zr-0	316.5 ± 16.3^a^	306.1; 326.9
Zr-30	267.6 ± 11.4^b^	257.2; 278.0
Zr-60	376.1 ± 16.8^c^	365.7; 386.5
Zr-120	311.5 ± 22.1^a^	301.1; 321.9
Zr-300	241.0 ± 12.9^d^	230.6; 251.5

p = 0.05. Different superscript letters indicate statistically significant difference.

The means and standard deviations (SD) of the contact angles of the experimental groups in contact with distilled water and diiodomethane, the dispersive and polar energies, and the resulting surface free energy of each group are described in Table 4. All experimental groups presented predominantly hydrophilic behavior, with a reduction of the contact angle with polar liquid (water) and an increased polar component and SFE.

**Table 4:** Mean contact angles and standard deviations for water and diiodomethane, polar (γp, in mN/m) and dispersive (γd, in mN/m) components, and respective SFE (γT, in mN/m) of the evaluated control and experimental groups of zirconia

Groups	Mean contact angle		Components (mN/m)		
	Water	Diiodomethane			
	Mean (SD) (º)	Mean (SD) (º)	γp (SD)	γd (SD)	γT (SD)
Zr-0	75.8 (0.8)	38.0 (1.1)	10.1 (0.37)	41.1 (0.5)	52.2 (0.5)
Zr-30	50.3 (1.2)	35.4 (0.4)	22.2 (0.6)	42.2 (0.2)	64.4 (0.6)
Zr-60	50.6 (0.7)	38.6 (2.1)	22.4 (0.4)	40.8 (0.9)	63.2 (0.8)
Zr-120	59.2 (0.2)	36. (0.4)	17.9 (0.1)	41.7 (0.2)	59.5 (0.2)
Zr-300	56.7 (0.8)	33.6 (0.6)	18.8 (0.4)	43.0 (0.2)	61.7 (0.4)

### Shear Bond Strength

Failure analysis showed that for all samples, adhesive or predominantly adhesive failures occurred at the interface between composite cement and zirconia. Table 5 presents the descriptive and inferential statistical analyses, with means ± SD, and 95% CI of the samples from the groups tested 24 h after cementation and after thermocycling for the “deposition time” parameter.

**Table 5:** Shear bond strengths of composite cement to zirconia in MPa

Groups	Thermocycling Yes/no	Mean and SD	95% CI
Zr-0	no	14.9 ± 5.0^B^	(11.2–18.6)
Zr-30		24.8 ± 5.0^A^	(21.1–28.5)
Zr-60		22.1 ± 8.5^AB^	(18.4–25.8)
Zr-120		23.0 ± 2.5^A^	(19.3–26.7)
Zr-300		20.1 ± 6.4^AB^	(16.4–23.8)
Zr-0 t		1.0 ± 0.7^c^	(0.2–2.1)
Zr-30 t		3.9 ± 0.7^b^	(2.8–5.3)
Zr-60 t	yes	3.6 ± 0.8^b^	(2.6–5.5)
Zr-120 t		6.1 ± 1.8^a^	(4.6–10.5)
Zr-300 t		5.6 ± 1.8^a^	(2.9–8.1)

Descriptive statistics (mean, SD, and CI, in MPa) and Tukey’s test of the groups before (24 h) and after thermocycling, p = 0.05. Different superscript uppercase letters indicate statistically significant differences between and among the groups after 24 h of cementation. Different superscript lowercase letters indicate statistically significant differences after aging.

For the group subjected to SBS testing 24 h after cementation (non-aging), one-way ANOVA revealed a significant interaction effect, indicating that the shear bond strength of the material changed due to the deposition time (p < 0.05). Zr-0 had the lowest shear bond strengths (14.9 ± 5.0 MPa), being statistically significantly different from the experimental groups Zr-30 and Zr-120 (24.8 ± 5.0 MPa and 23.0 ± 2.5 MPa, respectively). All experimental groups were statistically similar.

For the groups subjected to SBS after aging, one-way ANOVA also showed a significant interaction effect, indicating that the SBS of the material changed due to the deposition time (p < 0.05). The results analyzed by Tukey’s post-hoc test showed differences between and among the groups. Zr-0 t presented the lowest SBSs (1.0 ± 0.7 MPa), being statistically significantly different from the experimental groups. Zr-30 t was statistically similar to Zr-60 t, Zr-120 t, and Zr-300 t.

## Discussion

This study evaluated the effect of PECVD of SiH_4_ on the surface topography of HT zirconia as well as the chemical bonds achieved by composite cement, before and after aging, at different times (30 s, 60 s, 120 s, and 300 s), high voltage, and low vacuum. According to the results, the null hypotheses were rejected: the proposed surface treatment modified the chemical composition and changed the surface topography (which was dependent on the deposition duration and contributed to mechanical interlocking) of the HT zirconia, and changed the SBS of composite cement depending on the duration of SiH_4_ deposition, before and after the thermocycling, when compared with groups without deposition (Zr-0 and Zr-0 t).

Several studies have attempted to apply plasma treatments to create a chemically functionalized surface – due to the reduction of carbon-based contaminants – possibly creating hydrophilic surfaces and increasing SFE.^25,33,51,52^ This is consistent with the findings of this study, which showed that the longer the deposition duration of the ionized forms of SiH_4_ on the zirconia, the greater the amount of silicon on the surface (Table 2 and Fig 7). However, some early studies^33,51^ did not address the long-term bonding performance of the interfaces, and thus did not provide complete information about the real potential of new surface treatments for bonding zirconia.

The results obtained by XRD (Fig 2) showed that, on the surface of the zirconia after PECVD, the interaction between silicon and zirconia did not form a crystalline phase, showing peaks indicative of tetragonal ZrO_2_ alone.^27,41,48^ In support of these results, the FTIR spectra presented a broad band between 500 cm^-1^ and 700 cm^-1^ in all samples (Fig 3), a strong indication that the tetragonal phase was dominant.^7,27,48^ Also, no signs of a zirconia phase change were seen.

Figure 3 further shows the variations in the shape and position of the center of the band as a function of silicon deposition durations. The compressive stress shifts the center of the band to lower frequencies, and the tensile stress shifts the band to higher frequencies.^48^ These data agree with the results of XPS analyses showing that the Si-2p peak spectrum is at approximately 103 eV for Zr-30 and Zr-60 and 99 eV for Zr-120 and Zr-300 (Fig 7). Therefore, the increased silicon on the zirconia surface forms chemical bonds with oxygen (SiO_2_) and yields surface compressive stresses (as observed for Zr-30 and Zr-60). For Zr-120 and Zr-300, a strong peak at 99 eV indicates a high percentage of Si. A plausible explanation for this finding may be the continuous deposition of Si, forming SiO because of the interaction between Si and the SiO_2_ already present on the zirconia surface.^21^ This refers to silicon, which is highly reactive with the oxygen of the environment, forming SiO_2_ on the surface. Therefore, in XPS, we can observe both the oxygen and silicon peaks (Fig 7). The presence of SiO_2_ may have been responsible for the increased surface polarity^16^ and the increased surface energy values when compared with those of the Zr-0 group, indicating an improvement in surface wettability and reactivity with composite cement (Table 4).

PECVD also produced topographic changes on the zirconia surface. In the Zr-30 and Zr-60 samples, the bombardment with and penetration of silicon into the microstructure generated zirconia grains with irregular polyhedral shapes and a previously non-existent volume on the surface, as observed in FE-SEM micrographs (Figs 4 g-4i). As a consequence, compressive stress was also generated (Fig 3). The formation of silicon clusters and silicon cylinders occurred with the longest deposition durations (Zr-120 and Zr-300), resulting in high tensile stresses (Fig 3). The detachment of silicon cylinders from the microstructure (Figs 5 and 6) finally relieved surface compressive stresses, and the bands were then displaced to higher frequencies (Fig 3). Thus, it could be that the increase in SBS for Zr-120 and Zr-300 (before and after the thermocycling) was also due to the microretentive surface caused by silicon grain detachment (Figs 5 and 6). However, we must also consider that the amount of silica on these “120” and “300” specimens was higher than in the others. Moreover, this study showed that the mean surface roughness values for all experimental groups were lower than those in the Zr-0 group (316.5 ± 16.3 nm), which indicates a general tendency toward diminishing surface irregularities. Thus, an increase in the amount of silica with longer deposition durations might have been the most important factor responsible for the bonding results before and after thermocycling.

In this study, the SBS test was used, despite the arguments about unfavorable stress distributions at the bonding interface. This choice, however, was mainly based on it being a common, rapid, and easy-to-perform joint force test,^10,19^ extremely useful when zirconia is the bonding substrate. According to the SBS test, two consecutive chemical treatments (PECVD + Monobond N primer application) were able to produce a stronger bond of the composite cement, since the SBS was significantly higher for experimental groups than for groups in which primer alone (without PECVD) was applied (Zr-0 and Zr-0 t). The bonding mechanisms can be explained by: (1) the linking between the oxides (SiO bonds) on the zirconia and the universal primer containing silane methacrylate, (2) acting in concert with the methacrylate groups in the composite cement reacting with the methacrylate termination of the silane molecule.^3^

The SBS obtained in the Zr-0 group (14.91 ± 5.03 MPa) was higher than that of the Zr-0 t group (0.95 ± 0.70 MPa), showing a weak bond at the composite cement-zirconia interface with the use of a universal primer only. The same occurred for the experimental groups, which, before thermocycling, had nearly identical SBSs, but which showed a clear drop in bond strengths after aging (Table 5). The reduction in bond strength after thermocycling was possibly caused by degradation of the composite cement^53^ and hydrolysis caused by water at the composite cement-zirconia interface.^12,54^ The post-thermocycling results showed that adhesive strength was approximately three times higher for the Zr-30 t and Zr-60 t groups and six times higher for the Zr-120 t and Zr-300 t groups, compared with that of the untreated group (Zr-0 t). Therefore, the longer the deposition, the more numerous the binding sites and the higher the long-term bond strength.

In general, regardless of the treatment to improve bonding to zirconia, there is a steep decline in bond strength after aging. For instance, in the study by Ramos et al,^38^ which tested real interfacial bonding with a fracture mechanics approach and used traditional and silica-infiltration methods, composite cement/ceramic interface degradation occurred after thermocycling.

In contrast to airborne particle abrasion, PECVD does not produce major damage to the microstructure of zirconia surfaces. PECVD also enhanced the chemical adhesion of composite cement to zirconia, as opposed to previous results in which similar depositions were not succesful due to the low cohesive strength of the film.^36^ A thorough characterization of the zirconia showed silica clusters deposited on the zirconia surface, instead of a homogeneous film; this could be a limitation, as it can compromise bonding. Overall, it is an inexpensive method, but the equipment involved should be simplified to make it commercially viable. Future studies to compare physical treatments such as Al_2_O_3_ air abrasion or silicatization with the present method are warranted.

## Conclusion

The PECVD technique proposed in this work, with argon and SiH_4_ gas, was able to form a more reactive zirconia surface with a universal primer containing silane methacrylate and with composite cement. Also, the increase in deposition durations led to larger amounts of silicon on the zirconia surface, higher bond strengths after aging, and grain detachments. The SBS remaining after thermocycling was obtained with a minimally-invasive PECVD method. Finally, no phase transformation was associated with any of the PECVD deposition durations.

## Clinical relevance:

Developing and improving the PECVD technique could make it a new low-cost technology for internal conditioning of dental zirconia using a passive method to improve the chemical adhesion of composite cement to ceramics and dental substrates.
